# Effect of an oral supplementation with a proprietary melon juice concentrate (Extramel^®^) on stress and fatigue in healthy people: a pilot, double-blind, placebo-controlled clinical trial

**DOI:** 10.1186/1475-2891-8-40

**Published:** 2009-09-15

**Authors:** Marie-Anne Milesi, Dominique Lacan, Hervé Brosse, Didier Desor, Claire Notin

**Affiliations:** 1 Nutrition Department, Seppic (Air Liquide Group), Paris, France; 2 Bionov, Montpellier, France; 3 Isoclin, Chasseneuil du Poitou, France; 4 Behavioural and Cognitive Neurosciences, Université Henri Poincaré, Vandoeuvre-lès-Nancy, France

## Abstract

**Background:**

Recent studies have demonstrated a correlation between perceived stress and oxidative stress. As SOD is the main enzyme of the enzymatic antioxidant defence system of the body, we evaluated the effect of an oral daily intake of a proprietary melon juice concentrate rich in SOD (EXTRAMEL^®^) on the signs and symptoms of stress and fatigue in healthy volunteers.

**Methods:**

This randomized, double blind, placebo controlled clinical study was conducted with seventy healthy volunteers aged between 30 and 55 years, who feel daily stress and fatigue. They took the dietary supplement based on the melon juice concentrate (10 mg Extramel^® ^corresponding to 140 IU SOD per capsule) or a placebo one time daily during 4 weeks. Stress and fatigue were measured using four observational psychometric scales: FARD, PSS-14, SF-12 and Epworth scale. The study was conducted by Isoclin, a clinical research organization, located in Poitiers, France.

**Results:**

No adverse effect was noted. The supplementation with the proprietary melon juice concentrate bringing 140 IU SOD/day significantly improved signs and symptoms of stress and fatigue linked to performance, physical (pain, sleep troubles), cognitive (concentration, weariness, sleep troubles) or behavioural (attitude, irritability, difficulty of contact) compared to the placebo. In the same way, quality of life and perceived stress were significantly improved with SOD supplementation.

**Conclusion:**

This pilot study showed that an oral supplementation with a proprietary melon juice concentrate rich in SOD may have a positive effect on several signs and symptoms of perceived stress and fatigue.

## Background

In developed countries, people from all social classes are concerned about fatigue, stress and anxiety. It is estimated that 30% of the population will suffer from these symptoms at one point in their life [1]. It is known also that professional activity generates stress and fatigue, which are closely linked [1].

Several studies show that there is an emerging link between individual stress and intracellular oxidative stress. In a clinical trial on 42 women in the work force, a correlation between perceived stress and intracellular oxidative stress was demonstrated. In this trial, inflammatory markers like TNF-α or HbA1C were enhanced in women with a high burnout, indicating a role of inflammatory responses and oxidative stress in the pathophysiology of burnout [2]. In the same way, a clinical study on workers in a prehospital emergency service, showed a positive correlation between malondialdehyde (MDA), a biomarker of lipid peroxidation, and burnout levels [3]. Finally, a recent study on mice showed a positive correlation between the glutathione reductase activity and anxiety, suggesting that cellular oxidative stress seen in mice might be clinically translated as anxious and stressed behaviour in patients [4].

SOD is one of the main antioxidant enzymes found in living cells and organisms. Known since 1969 [5], SOD from erythrocytes was largely studied and used as a treatment for certain diseases like arthritis in a rat model [6] or in chronic radiotherapy damage in humans [7]. Since 2000, a proprietary melon juice concentrate containing high level of SOD has been developed, its use as a dietary supplement has been a new subject of interest and its antioxidant and anti-inflammatory properties have been demonstrated [8]. In healthy volunteers, the melon juice concentrate combined with wheat gliadin polymers, showed a protective effect against DNA damage resulting from oxidative stress induced by hyperbaric oxygen [9]. The same authors also showed that the melon juice concentrate rich in SOD combined with gliadin polymer reduced cell injury resulting from aortic cross-clamping in pigs [10]. Recently, it was demonstrated that Extramel^® ^(the melon juice concentrate combined with vegetable fat) prevents aortic lipids and liver steatosis in a diet-induced hamster model of atherosclerosis [11]. It seemed then very interesting to study the effect on perceived stress of EXTRAMEL^® ^(Bionov, Avignon, France), this melon juice concentrate containing high concentration of SOD which acts on oxidative stress at the cellular level.

The present pilot study aims to determine if a daily intake of this proprietary melon juice concentrate dosed to bring 140 IU of SOD is able to decrease the signs and symptoms of perceived stress and fatigue in healthy volunteers.

## Materials and methods

### Test Material

EXTRAMEL^® ^is a proprietary freeze-dried melon juice concentrate obtained by physical treatment (crushing of the melon, recovery of the pulp, centrifugation, filtration, freeze-drying) of a specific variety of melon (not a genetically modified organism) which contains high levels of SOD and other antioxidants (US Patent No 5 616 323). As SOD is very sensitive to oxygen, high temperature and acidity, the melon juice concentrate has to be coated. The product used in the study (EXTRAMEL^® ^microgranules) was coated with palm oil. The dietary supplements given to the volunteers were small hard capsules containing 10 mg Extramel^® ^(140 IU of SOD [12]) and starch for the *verum *(EXT) and starch only for the placebo (P).

The volunteers assigned by randomisation in two groups of 35 were given a food supplement (EXT) or a placebo (P) for 28 days: one capsule per day taken in the morning. The capsules were indistinguishable and were administered in a double blind approach.

### Methods: Psychometric tools

In order to evaluate the signs and symptoms of perceived stress and fatigue, the neurobehavioral sphere of the volunteers was explored. Moreover, their own perception of stress and its impact on their quality of life and sleep were evaluated. Four validated and well-known evaluation scales were then chosen:

1. ***Ferreri Anxiety Rating Diagram ***(FARD) [13,14] explores four poles divided in three items:

- Somatic pole (attitude, neurovegetative troubles, pain),

- Relational pole (apprehension, internal tension, difficulty of contact),

- Vigilance pole (irritability, sensorial troubles, sleep troubles),

- Cognitive pole (concentration, weariness, doubt-indecision),

with a total of 12 questions and a score from 0 to 72;

2. ***Cohen Perceived Stress scale ***(PSS-14) [15] evaluates the perceived stress during the last two weeks with 14 questions and a score from 14 to 70;

3. ***SF-12^® ^Health Survey ***[16] allows to measure eight aspects of the quality of life: general physical and mental health state (fatigue), physical and social functioning, physical and emotional health, pain and vitality. The SF-12 questionnaire is composed of 12 questions with a score from 3 to 38;

4. ***Epworth Sleepiness Scale ***[17] is a questionnaire intended to measure daytime sleepiness composed by eight questions with a score from 0 to 24.

The ***Beck Depression Inventory ***(BDI) [18], a 13 question multiple choice self-report inventory, which is one of the most widely used instruments for measuring the severity of depression, was used to avoid the inclusion of depressive volunteers.

### Study Design

The clinical trial was an intervention pilot study based on individual evaluation scales. The protocol followed was randomized, double blind and placebo controlled. It was approved by the Comité de Protection des Personnes Ouest III, the ethical committee of Poitiers, France under the number 2006-A00677-44 to be conducted by Dr Thierry Cantin.

A call for volunteers was made in the region of the investigation centre (Poitiers area, Poitou, 86, France) and the volunteers for the study were pre-screened by the investigator.

The inclusion criteria were to be between 30 to 55 years old, to have a BMI (Body Mass Index) ≤ 30, to have a stable professional activity for more than one year, to perceive a certain level of stress & fatigue, to be in full health, not taking any drugs or dietary supplements, not taking anti-stress or anti-fatigue drinks.

The exclusion criteria were to be pregnant or breast-feeding, to have a previous case of psychiatric disease, to have pathologies ongoing or active during the last month, to have received medical treatment (allopathic or homeopathic) during the previous month, to have taken a dietary supplement during the last month, to be in a stressful situation during the next month (wedding, birth, scheduled hospitalisation, important exam...).

Acceptable volunteers were called in for a screening and baseline evaluation using the five evaluation scales previously described: FARD, Epworth, PSS-14, SF-12 and BDI scale. Then, 70 volunteers were definitively included and participated in the clinical trial. These volunteers provided a written informed consent.

The volunteers were tested three times during a visit to the doctor. The first time was before the supplementation (D0), corresponding to the screening evaluation. A test was planned 7 days (D7) after taking the supplement and another one at the end of the trial (D28). The evaluation scales were filled out by the volunteers themselves. During every consultation, the doctor made a general clinical exam (arterial tone and cardiac frequency measure) and filled up an event journal with remarks on the volunteers, eventual undercurrent effects, the eventual pathologies and their associated treatment.

### Method of Screening the Volunteers

Axis were determined by combining the different scales:

• Physical Axis composed by FARD somatic pole (0 - 18), FARD vigilance pole (0 - 18) and SF-12 scale (3 - 38) with a total score from 3 to 74;

• Cognitive Axis composed by FARD cognitive pole (0 - 18) with a total score from 0 to 18;

• Relational Axis composed by FARD relational pole (0 - 18) with a total score from 0 to 18;

• Perceived Stress Axis composed by Epworth sleepiness scale (0 - 24) and Cohen's PSS-14 perceived stress scale (14 - 70) with a total score form 14 to 94. To be included, the volunteers had to get scores within the following parameters: BDI < 5; Physical Axis > 15/74; Cognitive Axis > 5/18; Relational Axis > 5/18; Perceived Stress Axis > 33/94.

The means of each score for the volunteers were: Physical Axis = 24.95; Cognitive Axis = 6.27; Relational Axis = 5.89; Perceived Stress Axis = 40.7.

### Statistical Analysis

The data were expressed as means ± SD of the evolution of the scores on each scale between D0 and D7 and between D0 and D28 in absolute and relative values. The paired Student's t-Test was used to analyse each group relative to the amplitude of variation between D0 and D7 and D0 and D28 and to compare results between groups at D0, D7 and D28.

## Results

### Study Population

Seventy volunteers aged between 30 and 55 years (mean 40.26) with a BMI from 17 to 42 (7 volunteers were included although their BMI was over 30) were enrolled and randomized into two test groups and no subject was dropout. There was no statistical difference between the two groups at baseline in scores for age, ethnicity, ...

During the 4 weeks of the study, the doctor filled up a file of events and no adverse effect was noted. Scores used for the statistical analysis are reported in Tables 1, 2, 3, 4, 5 and 6. The two groups are not equivalent in their base level score but this difference is not statically different except for vigilance pole, irritability and apprehension (data not shown) items of the FARD scale for which the scores are worst for the EXT group.

**Table 1:** FARD poles: scores at D0, D7 and D28 and evolution between D0 and D28 for P and EXT groups

	**Poles scores**						**Evolution between D0 and D28**					
	***D0***		***D7***		***D28***		***P group***			***EXT group***		

	***P group***	***EXT group***	***P group***	***EXT group***	***P group***	***EXT group***	***t student***	***p value***		***t student***	***p value***	

*Somatic pole*	4.78 ± 3.62	3.72 ± 2.64	2.40 ± 2.93	1.67 ± 2.32	1.75 ± 2.27	0.67 ± 1.24	3.280	0.003	**	5.672	0.000	***
*Vigilance pole*	5.34 ± 3.12	3.69 ± 2.56	2.96 ± 2.61	1.85 ± 1.88	2.79 ± 2.92	1.00 ± 1.24	2.541	0.018	*	3.813	0.001	***
*Cognitive pole*	6.38 ± 3.03	6.16 ± 2.42	2.16 ± 2.53	1.65 ± 2.26	2.13 ± 2.42	0.83 ± 1.29	5.992	0.000	***	12.710	0.000	***

**Table 2:** FARD poles: transverse comparisons between P and EXT groups at D0, D7 and D28

	***D0***			***D7***			***D28***		
	***t student***	***p value***		***t student***	***p value***		***t student***	***p value***	

*Somatic pole*	1.342	0.185	NS	1.004	0.320	NS	1.979	0.050	*
*Vigilance pole*	2.323	0.023	*	1.770	0.083	NS	2.701	0.011	*
*Cognitive pole*	0.319	0.751	NS	0.754	0.454	NS	2.225	0.032	*

**Table 3:** FARD items: scores at D0, D7 and D28 and evolution between D0 and D28 for P and EXT groups

	**Items scores**						**Evolution between D0 and D28**					
	***D0***		***D7***		***D28***		***P group***			***EXT group***		

	***P group***	***EXT group***	***P group***	***EXT group***	***P group***	***EXT group***	***t student***	***p value***		***t student***	***p value***	

*Attitude*	1.69 ± 1.55	1.53 ± 1.19	0.96 ± 1.54	0.63 ± 0.79	0.63 ± 1.06	0.17 ± 0.51	2.542	0.010	**	4.675	0.000	***
*Pain*	1.81 ± 1.51	1.25 ± 1.39	1.00 ± 1.29	0.52 ± 1.01	0.79 ± 1.14	0.17 ± 0.51	2.235	0.035	*	4.189	0.001	***
*Irritability*	2.38 ± 1.26	1.56 ± 0.95	1.40 ± 1.19	0.89 ± 1.01	1.13 ± 1.19	0.39 ± 0.78	3.340	0.003	**	4.123	0.001	***
*Sleep troubles*	1.94 ± 1.79	1.28 ± 1.46	1.28 ± 1.67	0.74 ± 1.23	1.33 ± 1.88	0.39 ± 0.50	1.234	0.230	NS	1.686	0.110	NS
*Difficulty of contact*	1.50 ± 1.05	1.53 ± 0.67	0.76 ± 0.83	0.26 ± 0.66	0.67 ± 0.76	0.28 ± 0.57	3.019	0.006	**	5.236	0.000	***
*Concentration*	2.25 ± 0.98	2.19 ± 1.09	0.92 ± 1.26	0.56 ± 0.97	0.79 ± 1.28	0.28 ± 0.57	5.027	0.000	***	6.761	0.000	***
*Weariness*	2.13 ± 1.48	2.00 ± 1.50	0.68 ± 0.95	0.37 ± 0.74	0.75 ± 0.99	0.17 ± 0.38	4.164	0.000	***	8.018	0.000	***

**Table 4:** FARD items: transverse comparisons between P and EXT groups at D0, D7 and D28

	***D0***			***D7***			***D28***		
	***t student***	***p value***		***t student***	***p value***		***t student***	***p value***	

*Attitude*	0.451	0.653	NS	0.983	0.330	NS	1.854	0.072	t
*Pain*	1.549	0.127	NS	1.501	0.140	NS	2.380	0.023	*
*Irritability*	2.909	0.005	**	1.672	0.101	NS	2.280	0.028	*
*Sleep troubles*	1.603	0.114	NS	1.333	0.189	NS	2.351	0.026	*
*Difficulty of contact*	0.142	0.887	NS	2.422	0.019	*	1.887	0.066	t
*Concentration*	0.241	0.811	NS	1.174	0.246	NS	1.741	0.091	t
*Weariness*	0.336	0.738	NS	1.320	0.193	NS	2.637	0.010	**

**Table 5:** PSS-14, SF-12 and Epworth scales: scores at D0, D7 and D28 and evolution between D0 and D28 for P and EXT groups

	**Scores**						**Evolution between D0 and D28**					
	***D0***		***D7***		***D28***		***P group***			***EXT group***		

	***P group***	***EXT group***	***P group***	***EXT group***	***P group***	***EXT group***	***t student***	***p value***		***t student***	***p value***	

*PSS-14*	36.88 ± 7.95	34.00 ± 6.03	30.88 ± 9.47	27.04 ± 8.06	30.17 ± 8.90	23.61 ± 6.58	3.975	0.001	***	5.767	0.000	***
*SF-12*	17.09 ± 5.46	15.38 ± 4.53	12.52 ± 4.90	11.52 ± 4.27	11.52 ± 4.96	8.89 ± 3.29	4.570	0.000	***	4.001	0.001	***
*Epworth*	8.03 ± 3.55	7.66 ± 3.43	6.28 ± 4.25	6.48 ± 3.77	5.67 ± 4.55	6.17 ± 4.64	3.582	0.002	**	1.971	0.065	t

**Table 6:** PSS-14, SF-12 and Epworth scales: transverse comparisons between P and EXT groups at D0, D7 and D28

	***D0***			***D7***			***D28***		
	***t student***	***p value***		***t student***	***p value***		***t student***	***p value***	

*PSS-14*	1.630	0.108	NS	1.580	0.120	NS	2.714	0.010	**
*SF-12*	1.371	0.175	NS	0.787	0.435	NS	2.037	0.049	*
*Epworth*	0.430	0.669	NS	0.181	0.857	NS	0.350	0.729	NS

### FARD Scale

The statistical analysis on somatic, vigilance and cognitive poles showed that scores of these poles were significantly improved at D28 in both groups (Table 1). This clearly indicates that an important placebo effect is present in the study.

Nevertheless, when the scores of the groups are compared at D28, a significant difference between them appears (Table 2): the three poles are much more improved in the EXT group than in the P group with respectively 18% more for the somatic pole, 25% more for the vigilance pole, and 20% more for the cognitive pole.

Concerning the relational pole, its improvement is not significant in either group (data not shown).

The further analysis of each pole also gave interesting results. In the P group, all items were significantly improved from D7 (Table 3) excluding sleep troubles and doubt-indecision (data not shown). In the EXT group, all items were also significantly improved from D7, excluding only doubt-indecision, but contrary to the P group, the improvement of certain items continued to increase in D28.

At D28, the results showed that pain, irritability, sleep troubles and weariness scores were significantly improved in the EXT group as compared to the P group, being respectively, 30, 22, 38 and 26% higher (Table 4). Attitude, difficulty of contact and concentration items also are more improved in the EXT group than in the P group, being respectively, 26, 26 and 22% higher, but there is no significance, it is only a trend. All other items (neurovegetative troubles, sensorial troubles, apprehension, internal tension and doubt-indecision) scores were not significantly different at D28 between the EXT and P groups, and seemed then to be only improved by a placebo effect (data not shown).

### Perceived Stress Scale

**Figure 1:**
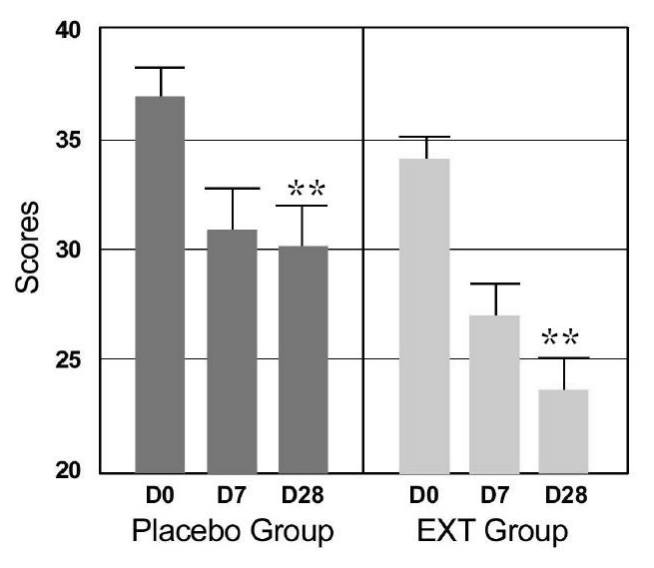
**Scores of PSS-14 scale (Mean ± SD) before, during and after supplementation**. EXT group: supplemented with melon juice concentrate rich in SOD. Transverse comparison between groups significantly different: ** p < 0.01

The analysis of the results of the PSS-14 scale (Table 5 and Figure 1) showed that a significant decrease in perceived stress occurred between D0 and D7 in both groups. However, after D7, in the P group, the placebo effect seemed to have reached its maximum level and perceived stress stopped to decrease, whereas in the EXT group, perceived stress continued to decrease with a statistically significant improvement of 12% (p = 0.01) compared to the P group at D28 and a global decrease of 30% (Table 6 and Figure 1).

### Quality of Life Scale

**Figure 2:**
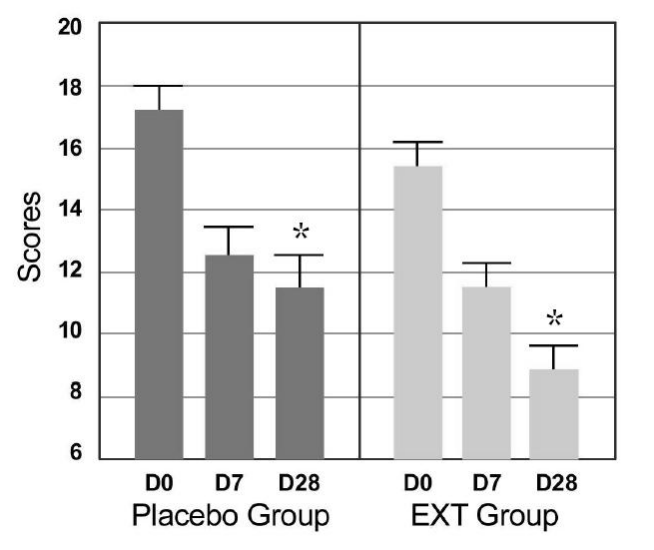
**Scores of SF-12 scale (Mean ± SD) before, during and after supplementation**. EXT group: supplemented with melon juice concentrate rich in SOD Transverse comparison between groups significantly different: * p < 0.05

The analysis of the results of the SF-12 scale (Table 5 and Figure 2) showed that a high and significant improvement in the quality of life occurred between D0 and D7 in both groups. However, after D7, in P group the placebo effect seemed to have reached its maximum level and the score stopped to decrease whereas in the EXT group, the quality of life continued to improve with a statistically significant difference of 9% (p = 0.049) compared to the P group at D28 and a global improvement of 42% (Table 6 and Figure 2).

### Sleepiness Scale

The analysis of the results of the Epworth scale (Table 5 and Table 6) showed non significant difference between the P and EXT groups.

## Discussion

The FARD scale showed that the symptoms linked to performance, physical (pain, sleep troubles), cognitive (concentration, weariness, sleep troubles) or behavioural (attitude, irritability, difficulty of contact), were visibly improved by the supplementation with the proprietary melon juice concentrate rich in SOD compared to placebo, whereas psychological behaviour (neurovegetative and sensorial troubles, internal tension and doubt-indecision) was not improved over the placebo effect. Perceived stress and quality of life (quality of life being an evaluation of both physical and cognitive fatigue) were also improved by this supplementation with the melon juice concentrate rich in SOD compared to the placebo, whereas no effect was shown on sleepiness.

Fatigue and stress were not very high in the population of the study and corresponded to a normal everyday level of fatigue and stress of working people in developed countries.

Because of this low base level of stress and fatigue, the placebo effect seemed to be very high whereas with a higher base level the difference between supplemented and placebo groups might be more significant. Nevertheless the study highlighted that the placebo effect was only present during the first 7 days of supplementation and not beyond.

It was interesting to note that, despite the high placebo effect and the low base level of stress and fatigue of the volunteers, the study highlighted that supplementation with the melon juice concentrate rich in SOD had a significantly higher effect compared to placebo on most of the variables of the study and tended to have a significantly higher effect on some others. This may indicate that this specific supplementation has an effect on signs and symptoms of perceived stress and fatigue covering physical and mental health such as pain, irritability, sleep troubles, weariness, perceived stress and quality of life; moreover it has a probable effect on others such as attitude, concentration and difficulty of contact; and it has no effect on signs and symptoms linked to relational behaviour and sleepiness.

Finally, it was interesting to discover that the supplementation with the melon juice concentrate rich in SOD has a significant effect on sleep troubles but not on sleepiness, which could be a side effect of many sedatives. This result added to the positive results on perceived stress, quality of life which evaluates physical and cognitive fatigue and overall physical and cognitive performances could suggest that Extramel^® ^supplementation acts as a cognitive and physical tonic.

## Conclusion

In conclusion, this pilot clinical trial highlights that an oral supplementation with EXTRAMEL^®^, bringing 140 IU of SOD, which is known to have an antioxidant activity on cellular level and fighting against oxidative stress, could have a positive effect on several signs and symptoms of perceived stress and fatigue, and particularly perceived stress, quality of life (physical and mental health condition) and some aspects of neurobehaviour. Further studies with a larger number of volunteers and a longer duration would be interesting to confirm these effects and better understand the action of an oral intake of this proprietary melon juice concentrate rich in SOD on stress and fatigue.

## Competing interests

This research was supported by Seppic (Air Liquide Group), Paris, France and Bionov, Avignon, France. The consultation and manuscript preparation were also funded by Seppic and Bionov.

## Authors' contributions

MAM and DL conceived the study and participated in the design of the study. HB designed and coordinated the study. DD did the statistical analysis. MAM, DL and CN drafted the manuscript. All authors read and approved the final manuscript.
